# “Anyone who weighs up risks doesn’t belong here”: how do elite handball players manage physical health risk throughout their professional careers?

**DOI:** 10.3389/fspor.2025.1553948

**Published:** 2025-04-17

**Authors:** Jan Bursik, Jochen Mayer, Ansgar Thiel, Felix Kühnle, Jannika M. John

**Affiliations:** ^1^ Institute of Sports Science, Eberhard Karls Universität Tübingen, Tübingen, Germany; ^2^ Institute of Health Sciences, University of Education Schwäbisch Gmünd, Schwäbisch Gmünd, Germany; ^3^ President's Office, German Sport University, Cologne, Germany; ^4^ Institute of Sport Science, Technical University of Darmstadt, Darmstadt, Germany

**Keywords:** health risk, risk management, qualitative, elite athlete, elite handball, reflexive thematic analysis, pain, injury

## Abstract

**Objectives:**

Elite athletes frequently encounter physical health risks, such as an injury, illness or pain, which are accompanied by sociocultural norms, individual perceptions, and situational pressures. While research has explored risk management in sports, limited attention has been given to the subjective experiences of athletes managing these risks across their careers. This study addresses this gap by examining how elite handball players manage physical health risks, focusing on the role of risk perception, evaluation, and coping.

**Methods:**

Using a constructivist lens, 11 handball players from the German national teams (5 females, 6 males) participated in biographical mapping interviews, providing insights into their career-long management of physical health risks.

**Results:**

Using reflexive thematic analysis, we generated four themes: (1) Externalizing risks and refraining from proactivity, (2) Relinquishing control under medical uncertainty, (3) Fluctuating prioritization of health or success, and (4) Calculated health-risk taking to achieve success. The findings illustrate that athletes’ risk management strategies vary based on career stage, injury experiences, situational priorities, and social pressures. The insights contribute to a deeper understanding of the dynamics of physical health risk management in high-performance sports and their sociocultural underpinnings. The study highlights the need for interventions that foster proactive risk management, emphasize personal agency, and balance performance with long-term health.

## Introduction

In the realm of elite sports, athletes regularly face situations containing varying degrees of physical health risks, which are often viewed as inherent to the competitive environment (1, 2). Physical health risks arise in situations where athletes’ decisions and behaviors have the potential to impact their physical well-being. These situations are typically characterized by an injury, illness or related symptoms such as pain, which may disrupt the athletic pathway (3) and require adapted behaviors (4). In response to a health-event, athletes often rely on quick fixes (cf. 5) instead of engaging in preventive measures, conducting rational risk assessments, or consulting with medical professionals. As a result, they participate in health-risk behaviors to maintain their social functioning in sport (6). These behaviors include playing hurt (7–9), using painkillers (5, 10), or shortening recovery times, which increases the likelihood of further injury (11), and potentially results in more severe or overuse injuries (cf. 12). These immediate behavioral solutions seem to be shaped by a variety of individual psychological, social, and environmental factors (5, 9, 13) such as the heterogeneous attitudes and expectations of athletes, coaches, and medical staff, but also cultural norms and values with regard to health risk management within the elite sports environment. These cultural norms have been shown to be embedded in a broader performance-driven framework, where success and competition often take priority over long-term well-being (14). Studies indicate that the performance narrative inherent to elite sports frequently overshadows considerations of health, recovery, or personal development, reinforcing an expectation that pain and injury are acceptable sacrifices for success (15–17).

A well-established body of sociologically informed research within elite sports has contributed to our understanding of how athletes manage physical health risks (1, 18–21). Using the heuristic of a “culture of risk”, these studies illustrated that athletes feel pressured to tolerate health risks, minimize or hide injuries, and engage in potentially harmful behaviors to align with performance expectations. Based on this line of research, risk management in athletic contexts can be conceptualized by four interrelated facets: risk perception, risk evaluation, risky behaviors, and coping strategies. In this context, Schnell, Mayer (22) have examined how athletes perceive and evaluate long-term physiological and psychological risks, often leading to the acceptance of these risks in their pursuit of success. This acceptance frequently manifests in risky behaviors in which athletes normalize or conceal their pain such as performing hurt or taking analgesics (e.g., 23, 24). Further, classic studies by Nixon (1) and Roderick, Waddington (7) into pain, injury, and risk provide critical insights into the sociocultural factors that reinforce such risky behaviors, often framing them as a symptom of the sport-specific culture of risk (1) and as a result of a decision-making process (13). Moreover, Schubring and Thiel (25) explored the coping strategies elite adolescent athletes use to navigate specific health risks (i.e., growth-related injuries), identifying both behavioral approaches (i.e., active agency) and cognitive strategies (i.e., distancing or rationalization).

Despite these advancements, a critical gap remains in understanding how athletes subjectively experience and navigate physical health risks over the course of their careers. While prior research has largely assumed that athletes assess risk and predominantly prioritize performance over well-being (e.g., 5, 13, 20), less attention has been given to how athletes individually perceive and interpret risk, and how these perceptions evolve throughout their careers and shape risky behaviors and coping strategies. In this regard, recent studies highlight that athletes’ individual experiences of risk, health, and performance are highly contextual and shaped by personal and structural influences (e.g., 3, 17, 26), necessitating a more nuanced exploration of individual physical health risk management.

Given this gap, this study aims to advance the understanding of how elite handball players manage physical health risks throughout their professional careers. By examining the experiences of elite handball players from the German National Team, this research moves beyond static models of risk-taking to explore how risk management is shaped by evolving personal, social, and institutional factors over time. In doing so, this study contributes to a more comprehensive understanding of individual risk management in elite sport across different stages of athletes’ professional careers.

### Theoretical framing of physical health risk management

In this article, we define physical health risk management as an individual process that involves risk perception, risk evaluation, risky behaviors and coping strategies aimed at reducing uncertainties and supporting the achievement of objectives (27). Physical health risks are conceptualized as emerging from the interplay between the objective assessments of an adverse event—such as medical evaluations of injury severity, which are often regarded as “objective” in professional practice—and subjective evaluations of the event's desirability (28). Thus, we conceptualize risk to be inherently situational and dynamic, shaped by individuals’ perceptions, priorities, and the socio-cultural environment in which athletes find themselves.

When examining how athletes manage physical health risks, it is essential to engage with established risk management theories. Risk theories encompass diverse approaches, with increasing recognition of the role of subjective interpretations. Scholars increasingly acknowledge that risk management is deeply shaped by the subjective perceptions of “objective” risk assessments (e.g., 28). In elite sports, athletes’ subjective perceptions are particularly consequential, as they directly influence subsequent behaviors, such as rehabilitating physical injuries or potentially exacerbating them by continuing to perform hurt.

### A constructivist approach to risk perception and risk evaluation

A constructivist perspective on risk management suggests that athletes continuously reinterpret and redefine risks based on career stage, external pressures, and personal priorities. Thus, it conceptualizes risk management in elite sport as a dynamic and evolving process, challenging previous research that treats risk perception and management as separate, stable constructs. This perspective further extends prior research on the culture of risk by highlighting that risk is actively constructed and reshaped over time, rather than merely accepted or rejected. Thus, by adopting a constructivist lens, this research positions risk perception and management as an ongoing, recursive process, offering a novel perspective on how athletes manage shifting perceptions of health risks in response to changing social and professional contexts.

In this context, Boholm and Corvellec (29) postulate a Relational Theory of Risk for understanding how individuals perceive and evaluate risk. According to their theory, risk perception is rooted in *situated cognition*, where individuals establish a relationship between a risk object (e.g., physical pain) and an object at risk (e.g., physical health or career progression). For a risk to be perceived, this relationship must be seen as causal, contingent, and the outcome for the object at risk must be undesirable. In the context of elite sports, risk objects like pain and injury are not inherently perceived as risks by athletes. They become tangible risks to athletes only when athletes associate them with threats to valued outcomes, such as career progression or physical health. Thus, according to Boholm and Corvellec (29), athletes would only consider playing with an injury as risky if they believe that playing hurt could potentially harm something they value. More specifically, for athletes to perceive a physical risk when playing with an injury, two things must happen: first, they need to recognize a causal and contingent relationship between playing with the injury and potential long-term health issues; second, they must view those long-term health problems as a significant and undesirable outcome in that given moment (cf. 30). However, if athletes believe that not playing with an injury would lead to losing their position on the team or missing out on key career opportunities (9, 31), they might see greater risk in not playing hurt. Thus, athletes juggle multiple objects at risk—health vs. career progression—and their perception of risk is shaped by which outcome feels more immediate or important at the time.

Relatedly, Corvellec (32) argues that risk perception is neither static nor purely culturally imposed, it is actively constructed and reconstructed. Consequently, athletes’ perceptions of risk may shift throughout their athletic careers as they define and redefine what they value most—be it health, career progression, or social status (cf. 33). An object is considered “at risk” only when it is explicitly or implicitly ascribed value, which in turn influences athletes’ behavior. This valuation highlights that athletes’ perceptions of risk are shaped by what they, or their environment, deem important at a given moment (cf. 32). In elite sport, the environment's social expectations and internalized norms particularly shape the athletes’ attitudes toward performance and well-being (1, 6, 34) and, consequently, their approach to risk. The use of the Relational Theory of Risk as the theoretical framework of this work allows to address the underlying individual cognitive processes involved in risk perception and evaluation, which supplements studies informed by the “culture of risk” heuristic. Instead of viewing risk solely as a cultural phenomenon, the principles of the Relational Theory of Risk suggest that athletes actively shape their understanding of risk. They constantly reassess what is “at risk”, such as immediate performance or long-term health, based on their career stage, personal values, and changing situational pressures.

### Individual dynamics of risk management in decision-making situations

Once individuals have perceived risks, the subsequent risk management strategies are strongly informed by the specific decision-making situation. General research on risk management strategies (e.g., 35–37) commonly presumes that individuals engage in risky behaviors when “an activity [is] carried out by people with a frequency or intensity that increases the risk of disease or injury” (36). While this conceptualization of risk behaviors is mainly objective, the research also investigates how people subjectively compare risk consequences and why they engage in subsequent coping patterns (e.g., 35, 38). Referring to Huber's (35) Theory of Active Risk Management, athletes seem to manage risky situations by employing risk-defusing operators (RDOs; 31). These operators involve additional actions or cognitions which reduce the perceived risk and thereby enable athletes to choose the subjectively most promising alternative. Such individual risk management strategies, as researched in a quasi-naturalistic scenario study by Mayer, Burgess (31), are used by athletes to minimize the perceived risk by defusing either the probability of potential sporting consequences (e.g., losing a game) or medical consequences (e.g., worsening the injury).

For example, an athlete with a ligament rupture might identify two RDOs to reduce sporting consequences. One involves playing hurt; the other entails assessing the opponent as weak enough for the team to win without them. While both strategies attempt to reduce the perceived competitive risk, they conflict in terms of behavioral management (i.e., playing hurt vs. sitting out). To make a decision, the athlete must also consider the medical risks—such as worsening the injury—and may adopt an additional RDO, such as stabilizing the ligament with tape. This creates a subjective sense of control, allowing the athlete to justify competing hurt. However, this perceived control does not necessarily reduce the actual risk (32); even if the athlete feels protected, the danger of aggravating the injury remains. Overall, the process of identifying RDOs, weighing different consequences and choosing the most promising alternative as described in the Theory of Active Risk Management (31, 35) highlights the need for investigating subjective risk management.

A constructivist perspective on risk perception and evaluation, combined with active behavioral risk management, offers a novel contribution to the research of athletes’ physical health risk management. Following the assumptions delineated within our theoretical approach, risk perception, risk evaluation, and behavioral coping with physical health risks are interwoven, fluid, and highly contextual. The use of RDOs illustrates how athletes subjectively construct and negotiate multiple competing risks (e.g., performance vs. health) rather than making binary decisions about how to behaviorally cope with physical health risks. This perspective on the individual dynamics of risk management moves beyond existing discussions and scholarship by highlighting that risk management is not solely a socialized behavior but also an evolving, self-regulated process in which athletes construct risk perceptions and evaluations and strategically modulate their behavioral responses over time.

### Purpose of the study

Despite the established body of sociological research surrounding risk acceptance and normalization in the culture of risk (1, 18–21) and a growing body of research examining the dynamics of “risk-behaviors” in elite sport (9, 10, 12), a notable gap remains in our understanding of how athletes actively construct, evaluate, and behaviorally cope with physical health risks over the course of their careers. While theories such as the Relational Theory of Risk (29) emphasize the subjective and context-dependent nature of risk perception and evaluation, it has not been applied to the elite sport context and thus, does not fully account for how athletes have to evaluate competing priorities, such as health, career progression, or status. Similarly, frameworks like the Theory of Active Risk Management (35) focus on risk management strategies but pay less attention to athlete's subjective risk perception. Consequently, while both frameworks provide valuable theoretical foundations into sub-aspects of risk management, they have yet to be fully integrated to capture the dynamic and interwoven nature of physical health risk management in elite sport.

To address this gap, this study adopts a constructivist lens to examine how elite handball players adapt their risk perceptions, reinterpret their risk evaluations, and actively shape their behavioral coping strategies with risks throughout their careers. Unlike prior research, which has primarily examined sub-facets of risk management or considered risk-taking primarily as a product of socialization (1, 6, 31), this study primarily focuses on the agency of athletes in constructing and modifying their physical health risk management in response to shifting personal and contextual factors.

This study employs a qualitative biographical study design to provide an in-depth and longitudinal perspective on how athletes individually construct and adapt their cognitive and behavioral strategies for managing physical health risks across different career stages. By capturing the experiences of elite handball players, this research examines how risk perception, evaluation and coping strategies evolve as athletes navigate shifting career demands, personal priorities, and contextual pressures.

## Methodology

Constructivist theory posits that individuals do not passively absorb information from their environment; instead, they actively engage with and interpret their experiences to construct personal meaning and knowledge (39). Thus, we assume that elite athletes subjectively perceive and evaluate risks, which in turn shapes how they subsequently behaviorally manage physical health risks.

### Participants and data collection

We utilized a purposive sampling approach aimed at deepening the understanding of the phenomenon (40) in a highly elite sample which is “most likely to yield appropriate useful information (41). To increase feasibility and comparability, we limited the sample to the sport of handball while aiming for a variation in age and gender. Handball was selected because it offers an ideal platform for research on health-related risk behaviors due to its high injury incidences and risk-taking behaviors (9, 10, 42). At the time of the interview, the participants were part of the German handball national team (male or female). On the club level, all participants played in the *German Handball Bundesliga* (1st division). The mean age was 27.6 (range 22–34 years).

The interviews with eleven handball players (5 females, 6 males) were conducted by the first author and AR in 2022. Quiet locations that were easily accessible for the athletes were chosen for the interviews (such as their training facility). The average interview lasted 86.1 minutes (ranging from 50 minutes to 128 minutes). The study received ethical approval by the ethics committee of the Faculty of Economic and Social Sciences ethics committee at the University of Tübingen (AZ: A2.5.4-176_ns). Before the interviews, all participants signed informed consent forms.

For our interviews, we used the biographical mapping method to capture temporal dynamics of managing physical risks throughout careers (see 43 for further details). First, participants were asked to their professional career journeys, reflecting on significant life events, relevant developmental stages, and critical health-related events (see also: 44). Participants then engaged in a drawing activity coupled with a think-aloud protocol (43). Comparable to a therapeutic lifeline tool, this approach helps to “facilitate a structured recall of a sequence of previous events, particularly within the context of qualitative interviews” (cf. 45, p. 11). Athletes were asked to visually depict the intensity of health and performance-related experiences throughout their careers, as such nuances are often difficult to convey verbally due to their temporal and sensitive nature (cf. 46). The dimensions they were asked to draw over the course of their professional careers included pain intensity, the extent of painkiller use, health-related willingness to take risks, and perceived performance capacity. As they drew, participants were encouraged to verbally reflect on the curve courses (cf. 47). Relevant reflections about their behaviors surrounding health-related risks were verbalized and chronologically situated by the athletes throughout their professional careers. In addition, we also asked participants specific questions about the curves they drew. For example, athletes were asked to explain sharp increases in painkiller use or fluctuations in their perceived performance capacity, which provided further context and understanding of how they navigated critical moments in their careers. This verbal data was incorporated into our analysis. To guarantee anonymity to the participants, we used pseudonyms and deleted all identification information in the interviews. The biographical mapping method allowed us to capture how the athletes perceived and interpreted their past experiences regarding physical health risks, offering deeper insights into their subjective reality (48). The interviews were transcribed verbatim by a professional company.

### Reflexive thematic analysis

The transcripts and the audio tapes served as the raw data. The lead author corrected the transcripts through listening to the audio while reading the transcripts and familiarized himself with the data. To explore how the elite handball players managed physical health risks, the data analysis was conducted using an inductive reflexive thematic analysis (49). The adaptability of reflexive thematic analysis enabled us to develop our analysis inductively and identify underlying patterns of perceptions and described behaviors within the interviews. This proved to be a considerable methodological advantage, as the handball players seldom directly addressed how they managed physical health risks throughout their professional careers.

The data was analyzed in coherence with the six phases of reflexive thematic analysis (Braun & Clarke, 2021) and carried out using MaxQDA (Verbi Software, 2024). While becoming familiar with the data, the lead author marked sections of the interviews that seemed related to the management of physical health risks. This included instances where athletes described how they navigated injury risks during critical phases of their careers, such as deciding whether to compete in an important championship despite an ongoing or anticipated injury. The lead author also noted down his first analytical insights, such as recognizing patterns in how athletes assessed risks and balanced long-term health considerations and immediate performance goals.

After data familiarization, the first author mainly coded at the semantic level, staying close to the text (exemplary code: “Without a diagnosis I basically HAVE to play”). Throughout the process of coding (i.e., all interviews were coded three times), the codes became more latent (“Lack of Diagnosis pressures continued participation”). During the coding process, the research question was revised to better align with the data (49). Initially, the research question focused on how elite athletes retrospectively reflected on their physical health risks, it became clear that the data provided more insights into how handball players described their thoughts and perspectives on physical health risks in specific situations, as well as how they managed these risks. The lead author re-examined the codes to ensure they fitted with this new research focus. After the third round of coding, within MaxQDA, the first author grouped the codes into categories of codes focusing on overlaps in meaning (exemplary category: “Lack of Diagnosis as a relevant criterion for risk taking”). Then, he printed out the code categories and generated initial candidate themes with similar underlying meanings. Some categories (exemplary category: “Health risks must be taken for success”) were used as candidate themes whereas other categories (exemplary categories: “Lack of Diagnosis as a relevant criterion for risk taking” and “With clear diagnosis, a risk assessment becomes possible”) were merged to a new candidate theme (exemplary candidate theme: “The influence of diagnosis on risk management”). These themes were continuously discussed with the last author who acted as a critical friend (50). In this process, the themes were renamed and reorganized. In research meetings with all authors, the themes were presented and discussed. In this process, the first and last author defined the core ideas of each theme as well as its boundaries. The first author then went back to the related interview passages to ensure that the generated themes were supported by the original data. During this last stage, the themes were refined, and the analytic narrative was developed. The stages of generating initial themes and refining and reviewing potential themes overlapped, highlighting the iterative process inherent to reflective thematic analysis (51).

### Reflexivity and quality criteria

Aligned with our paradigmatic stance, we adopted a relativist approach to quality (52, 53) and selected quality criteria that reflect our paradigmatic position and study objectives: transparency, sincerity, resonance, worthy topic, and credibility. The lead author worked as a soccer coach in his free time and has also gathered experience as a soccer player himself experiencing various minor and overuse injuries. During the research process, he reflected on his behavior concerning injury-related risk-taking from his coach and athlete perspective. Additionally, his experience as an applied sport psychologist in handball helped him establish an emotional and empathetic connection with the participants. To challenge any interpretations and assumptions influenced by his own experiences with sports-related injury risks, he regularly consulted with critical peers and maintained a reflexive journal where he systematically recorded his emotional reactions to the interviews, reflections on how the interviews affected his perspective on risk-taking in sport, and evolving thoughts on the data and analysis process (50). These strategies ensured the transparency and sincerity of our study by emphasizing the impact of personal experiences on the research process and findings.

## Findings and discussion

We constructed four themes which illustrate how athletes manage physical health risks: (1) Externalizing risks and refraining from proactivity; (2) Relinquishing control under medical uncertainty; (3) Fluctuating prioritization of health or success; (4) Calculated health-risk taking to achieve success.

### Externalizing risks and refraining from proactivity

This theme highlights how athletes externalize risks by attributing injuries to factors such as chance or fate. As a result, athletes fail to recognize the relationship between the risk objects (e.g., pain) and the object at risk (e.g., injury) and do not engage in proactive behaviors to minimize “objective” risks, such as seeking medical care or modifying their load: “Something can always happen. (…) That's why. I don’t really think about the risk I’m taking (…). For example, with the patella tendon (…) I also thought to myself, well then it just ruptures” (Maya). In this quote, Maya described her belief that injuries can “always happen” and are outside of her personal control, discouraging her from actively thinking about and managing risks. This aligns with findings demonstrating that athletes who perceive less control over injury risk tend to engage in higher levels of risk-taking behaviors (54). This pattern of externalization was particularly evident early in athletes’ careers, when athletes did not interpret objective health risks (i.e., pain or physical strain) as warnings that would require action.

Accordingly, athletes tended to perceive injuries as sudden events, as exemplified by Jack: “Actually, it wasn’t that my performance was slowly declining day by day. No, my performance was still there, and then boom—one day you twist your ankle, and suddenly your performance is gone, just like that.” Adding to Jack's perspective, Tom emphasized the uncontrollable nature of injuries reinforcing the idea that injuries can occur randomly, regardless of an athlete's physical preparedness or prior physical stress:

It can happen without any prior strain—you can twist your ankle, or you could tear up your knee because you’re already worn out. In this sport, that's just how it is. If someone bumps into your knee, it doesn’t matter how trained or pre-injured you are, nothing can help you in that moment.

Tom's perception reflected a broader tendency among handball players to view impaired health, such as pain or illness, as no more likely to lead to an injury than a state of good health. This belief led athletes to overlook the significance of impaired health as a risk object and to neglect the need for proactive risk management to prevent potential physical consequences such as an injury (i.e., object at risk).

Moreover, athletes frequently recounted their behavior in objectively risky situations with a sense of fatalism, emphasizing how they “were lucky” to have avoided more severe consequences while disregarding the risk associated with the present health event. This can be seen in the following quote by Lisa: “I had patella problems for a long time. I don’t know how clever it was to keep playing with it all that time (…) but I was lucky that nothing worse happened.” Similarly, Tom attributed a favorable outcome after neglecting an objective physical health risk (i.e., playing with a shoulder injury) to luck: “I was really lucky that nothing worse happened, even though I had previous injuries.” Both athletes externalized risk by attributing their injury outcomes to luck. By framing injuries as unpredictable and beyond their control, they distanced themselves from acknowledging how their decisions and behaviors might have contributed to their physical condition. This perspective reinforced a passive approach to risk management, discouraging proactive behaviors such as medical intervention or load modification. By attributing injuries to external factors like chance or fate, athletes neglected the relationship between potential risk objects and the objects at risk. This externalization, in line with the Relational Theory of Risk, shaped their subjective perception to the extent that they sometimes did not perceive physical health risks at all (cf. 29).

Underpinned by a general normalization of pain and injury, as described in various sociological studies (1, 7, 21), athletes seemed limited in their ability to proactively manage physical health risks, which in turn reinforced and sustained this normalization. This can be seen in the following quote by Tom in which he described how he typically reacted to pain:

Yeah, because I’m the kind of person who really pushes himself to the limit, I don’t go to doctors very often when I’m in pain. I always try to avoid going to doctors (…). Doctors are not important to me. I only go to the doctor when nothing works anymore in my body.

Since he viewed injuries as events beyond his control, he did not consider it necessary to seek medical care when he was in pain. Without seeing a clear need for intervention, Tom's approach reflected the wider normalization of pain and injury (1, 21), expanding it to an individual tendency to disregard the relationship between proactive care and long-term consequences.

### Relinquishing control under medical uncertainty

Throughout their careers, athletes frequently relinquished control over their health decisions, downplaying symptoms and postponing action until a formal diagnosis validated the necessity for risk management. This reliance on external validation reflects compliance with cultural norms in elite sports, where enduring pain is expected (55), and symptoms are frequently dismissed as manageable without a clear medical diagnosis. Emily exemplified this phenomenon by reflecting on her experience with unaddressed pain symptoms: “Those are all things that, if I had been diagnosed earlier, I might have been able to react more quickly and simply shorten the period of pain—and maybe even the rehab phase.” This reliance on diagnosis is consistent with aspects of the Relational Theory of Risk proposed by Boholm and Corvellec (29), suggesting that an individual may not perceive a poor health state as a potential risk object unless it has been externally validated through a diagnosis. In the absence of a formal diagnosis, athletes engaged in selective risk assessment, often failing to recognize the connection between symptoms and potential long-term consequences. This dependency on diagnosis was further illustrated by Lisa's differentiation between injuries where something was “inflamed but not broken” vs. those where something was “really damaged,” requiring time to heal:

That's different for me because I wasn’t injured to the point where something was actually damaged. It's more like something was inflamed but not broken. With my other diagnosed injuries, something was really damaged, something that needed time to heal properly.

This mindset suggested that symptoms only became a recognized risk when labeled as such by medical professionals.

Medical uncertainty in the absence of a formal diagnosis often resulted in ambiguity for athletes, which in turn led to a blurred perception of injury risks. This was because the subjective categorization of symptoms as a serious health event (i.e., injury or illness) was limited by the lack of a clear objective diagnosis. Lisa exemplified this challenge as she articulated her perplexity over symptoms (i.e., pain in the Achilles tendon) that were not formally recognized as an injury, which in turn impeded her ability to take action: “It wasn’t a type of injury where you could say, “okay, this is an injury, and I need to take a break” (…). I didn’t even realize I was injured”. Lisa's excerpt demonstrated how diagnostic ambiguity can impair an athlete's perception of risk, leading them to underestimate symptoms and avoid proactive management. Consequently, athletes reported a lack of information to make independent health decisions. Olivia highlighted the challenge of self-assessment when faced with ambiguous symptoms. She illustrated a situation in which her coach noticed her pain and enquired as to her ability to continue playing:

“What do you think, can you or can’t you play?” This question is easy to ask, because you can’t expect the player to approach it objectively and say: “I can’t do it.” Everyone of us would say: “I can do it somehow.”

The inability to correctly assess their state of health often prompted athletes to defer responsibility for health decisions to coaches or medical professionals, thereby reducing their personal agency in managing physical health risks. As Paul, Jones (56) describe, athletes find themselves in a “position of vulnerability” (p. 3) when they are expected to correctly assess their potentially complex health status. Within our interviews, such a position of vulnerability often occurred when athletes had transitioned to senior levels of elite handball or had recently been nominated to a new team. Hanna's reflections on her first club in elite senior handball illustrate this issue: “Nobody knew what the current status was or what was on the doctor's report. And then the coach expects me as a player to tell her the medical point of view which I sometimes didn’t really understand myself”. As a result, athletes relied heavily on external authorities for guidance, which can also be seen in the quote by Maya: “Then the doctor says: “Yes, she has to take a week off.” (…) So, for me, it was always the case that I didn’t have much to say in the matter.” In this context, cultural expectations and diagnostic uncertainty fostered a coping strategy of relinquishing control, where athletes increasingly deferred to medical or coaching staff for decisions in risky situations.

Furthermore, the internalization of social and cultural pressures within elite sports often compelled athletes to relinquish control over their health by aligning with the norm to perform hurt, particularly when pain was not acknowledged as a severe injury. Lisa reported: “It [Tibial Periostitis] wasn’t really THAT kind of injury where you could say, “Okay, I have to sit out for this””. As a consequence, she performed while in pain. Similarly, Maya indicated her reluctance to take rest periods, despite experiencing pain: “I just always want to be there to play. And I wouldn’t sit out because I’m in a bit of pain. Yes, I couldn’t reconcile that with myself.” These statements emphasized how athletes internalized a cultural narrative that viewed enduring pain as an inherent aspect of their role and identity (9, 33), thereby limiting active risk management by downplaying the severity of the symptoms, particularly under conditions of medical uncertainty.

For many athletes, a formal diagnosis constituted a defining point that legitimized symptoms and validated the need for proactive risk management. Olivia described how receiving a diagnosis for a patellar injury changed her approach to her condition: “In general, I find it hard to cancel a training session, and almost impossible to miss a game—unless there's a real diagnosis.” In this context, Olivia underscored how a diagnosis enabled her to reframe her pain as a legitimate injury, thereby allowing her to adopt a more careful approach to her recovery and thereby to actively manage risk. Steve echoed this stating, “It's definitely easier when it's clear [the diagnosis]—when you know, “I can't play today, it's just not possible.”” Thus, while athletes often dismissed pain, an official diagnosis reframed pain as a legitimate health risk, prompting athletes to reassess their risk management strategies. This findings highlights how athletes often undergo cognitive reappraisals after receiving a diagnosis (55), where the severity of an injury and the subsequent evaluation significantly alter their response to the injury (55). The phenomenon of diagnosis serving as a catalyst for behavior change has been scarcely researched in the elite sports context. However, it has been documented in medical fields as a critical turning point in managing symptoms and promoting self-care (e.g., 57, 58). This notable reliance on diagnosis illustrated how athletes were constrained within a system where both they and their support networks acknowledged symptoms as legitimate health risks only after they had been validated by medical authorities. Prior to such classification, athletes appeared to lack an understanding of the relationship between their symptoms (risk object) and potential health consequences (object as risk), which may have led them to continue risky behaviors despite the potential for deterioration in their physical condition.

### Fluctuating prioritization of health or success

This theme describes how athletes frequently fluctuated between prioritizing either health or success (cf. 17) when perceiving, evaluating, and managing risks. These shifts were influenced by social pressures, situational factors, and internal conflicts. Lisa demonstrated how her approach to physical health risks (i.e., pain or injuries) varied according to specific situational and contextual factors throughout her career:

It was quite different for each injury (…). For my shoulder, I had a very skilled physiotherapist, who would just say “yes” or “no”, and that took a lot of pressure off me. The coach didn’t really put any pressure on me either, so it was pretty relaxed. (…) But with the small muscle tear, things were a bit more complicated because I had a more important role in the team at that point, being one of the older players. By then, I knew my body better and could say, “I don’t feel ready yet.” I’d tell the physiotherapist, and she would pass that on. But with my Achilles tendon issues, I didn’t handle it very well. I think if I’d had a coach who told me, “Take it easy for a couple of weeks,” that would have been good. But I didn’t speak up because I was unsure if I was just complaining too much or if it was something real.

Lisa's approach illustrated how her focus moved back and forth between prioritizing self-care or performance (while suppressing injury concerns), contingent upon her role within a team and the external guidance or pressures exerted by coaches or management. This dynamic aligns with theoretical assumptions that posit that risk perception is a dynamic process which evolves according to context-specific values (29, 32).

External stakeholders, including coaches and medical staff, played a pivotal role in shaping athletes’ management of physical health risks, particularly with regard to their engagement in risky behaviors, a pattern that recurs in research on performing hurt (eg., 9). The handball players frequently followed the advice from these stakeholders when navigating health and performance. Emily recounted an instance when she obeyed to both her coach and her medical team, who persuaded her to compete despite a knee injury. However, she contrasted this with an incident where she prioritized her health due to good medical care, further underscoring how athletes’ risk management strategies are not fixed but shift based on situational factors such as evolving support systems:

Then, with the knee, it was shortly before the German Youth Championship Final. With tape, painkillers, and everything you can always manage another two games. The persuasion of the doctor and coach at the same time. That's why I didn’t think much about any health risk (…). Because that was the Final Four of the German championship. And according to the coach and staff, they couldn’t have played it without me. So, I listened to them, because I was still young, and I played that game. (…) In contrast, I received good medical care for the fracture. That's why it felt safe for me [not to play].

Emily's health risk assessment was continuously shaped by the attitudes of her coaches and the medical team, which emphasized either performance or health risks depending on the situation. For example, during the Championship final, she downplayed health risks due to her team's focus on performance and a strong sense of responsibility for her team, while with her fracture, the concern for health by her medical team led her to prioritize recovery. Similarly, Hanna noted that unlike previous coaches, her current coach aimed to create a balance between performance and health that was recognized and followed by the players:

He [the coach] demands a lot from us. Also, that we push ourselves to our limits which is good. But he clearly says: “It's better to take a break from training than to be injured for three weeks afterwards.” I like that.

Hanna's statement illustrated that external perspectives and social pressures influenced how she weighed health against performance, shaping whether she adopted a performance-focused or health-centered approach in a given situation which in turn impacted how she handled health risks (e.g., 56, 59).

Furthermore, athletes highlighted how situational factors were crucial in their consideration of the potential consequences of taking physical health risks (i.e., performing hurt). Emily, for instance, reported that she had engaged in performing hurt, particularly during important games at the end of the season: “Then came my knee injury. I had to play one more game. I did everything I could to play this game or, I had to do everything I could to play this [last] game.” Similarly, athletes felt pressured to step in when teammates were unavailable, even if it meant playing with pain and/or analgesics. Jack recalled a situation where he felt compelled to play with severe pain due to his teammate's injury: “Yes, actually, because I was alone in my position at the time. Because my partner [on the same position] had a cruciate ligament rupture at the time. That's why I had to play.” The repeated phrase “had to play” indicated a focus of both players on sporting and social consequences rather than health consequences (cf. 31).

The internal ambivalence between prioritizing health and achieving success also emerged as athletes reflected on health and risk, with the tension often intensifying during key career milestones, like becoming a starting player on their team. When asked to reflect on his risk-behavior throughout his career, Tom explained: “I always had in mind that I had to help the team, and I had to do my best for the squad. And accordingly, the team's success was always more important than my own health.” Conversely, he later emphasized the significance of physical wellbeing when questioned about his conceptualization of health: “Health is simply the greatest good. And you simply have to take care of yourself, of your body.” Tom's accounts demonstrated the fluctuating salience of health or success that often shifted throughout an athlete's career. Athletes may perceive physical pain (i.e., risk object) as a threat to their health (i.e., object at risk), particularly after having experienced severe injuries themselves or within their team. However, this focus was often deprioritized when it competed with other valued outcomes, such as contributing to team success or a feeling of collective responsibility and commitment to their team (cf. 9, 29)

While athletes often neglected objective risks in favor of performance, severe explicit health risks prompted a shift in risk evaluation, making health and career continuity more salient than short-term success. Emily recounted an experience where medical advice prompted her to withdraw from playing with a hand fracture: “Because it was a fracture, they told me that especially in defense, if I took too many hits, it could shift. That's when I knew I had to stop.” Emily highlighted that the clear warning from medical professionals marked a turning point, making the specific health risks in her situation more tangible and prompting her to adjust her risk management approach. The explicit risk of a more severe fracture shifted Emily's priorities, showing how some risks can bring health considerations to the forefront for athletes (cf. 9, 13).

### Calculated health-risk taking to achieve success

In this theme, we draw attention to how handball players demonstrated a belief that risk-taking was necessary to achieve success. From early in their careers, athletes acknowledged the physical risks (i.e., potential for injury) inherent in handball, showing a calculated willingness to accept any consequences. Hanna noted sarcastically: “We all know that handball is not exactly the gentlest sport on the body.” This characterization of handball was echoed by Lisa who similarly characterized the physical risks of handball: “In handball, you [meaning herself] always take a certain amount of risk because it's a very physical sport. That's why I'd say you [meaning herself] always have a fundamentally high willingness to take risks.” These remarks reflected the players’ perception that potential risks were an inherent aspect of professional handball.

Building on this awareness of the general physical health risks associated with handball, players also recognized the specific health risks involved in playing through particular injuries or pain. Consequently, they knew that injuries could deteriorate and have long-term consequences. Emily described how she continued to play with various injuries (e.g., hairline fracture or a partially torn labrum) despite being aware of how each injury could have led to more serious injuries:

I took risks playing with the hairline fracture. I definitely risked getting a bone fracture. I know that now. I knew that I could take one wrong step, and the bone would just break. With the knee injury, it already felt fatigued. I knew that even with tape and so on, you can still get a more severe injury than just a bit of fluid in the knee, like an ACL. With the hip, only the labrum was damaged. But playing with it could have also caused more damage than it did.

Emily's reflection, along with Hanna's general statement that “I think we are aware of specific risks” underscored a recognition of the risks that accompanied their decisions to play through pain and injury (c.f. 22).

Furthermore, it appeared that athletes’ awareness of these risks increased throughout their professional careers, especially after experiencing their first serious injuries. Lucas highlighted the progression of risk awareness throughout his career, noting: “Now you [meaning himself] pay more attention to your body (…). Now you understand your body more. At that earlier time (…) you thought that nothing could hurt you.” In this passage, he contrasted his perception as an older athlete with his earlier sense of invulnerability commonly seen among younger athletes (17).

However, the awareness of risks did not necessarily reduce risky behavior. Instead, with accumulated experience, players perceived that they had developed a stronger ability to manage their bodies when confronted with physical health risks, which in turn seemed to lead to increased calculated risk-taking. Olivia remarked how knowing the limits of her body emboldened her to push her boundaries even further: “I think I know my body well enough now to understand how much I can push it.” Lucas echoed this sentiment, acknowledging his increased confidence in managing physical risks: “I would take even more risks now. Because now I understand it better.” These statements suggest that, rather than promoting caution, experiences of risk management may have led athletes to feel more competent in handling future risks, reinforcing their awareness of risks and engagement in risky behaviors at the same time (cf. 22, 26).

Relatedly, athletes perceived physical risk taking (i.e., playing with an injury) as critical for success, which was clearly articulated by Tom:

And now for us, it's all about winning, it's all about being the best. And I think it's automatic that you simply increase the risk because it's all about everything. And we, for example, also knew that in the season if we all stay healthy or mostly healthy, the chances are very, very high that we will be successful. And that's why the risk just increased.

Tom's statement reflected a commitment to risk-taking in games in pursuit of success. Similarly, for Maya, tolerating pain was not only important during games, but she also perceived maintaining a rigorous training regime as necessary to achieve her goals: “When I'm in pain, I prefer to train because, well, you have goals, and you can only achieve them if you train.” Thus, the athletes not only acknowledged the physical health risks and willingly accepted the consequences (8, 22), but seemed convinced that the risks were necessary for achieving success at the elite level of sports.

Lastly, in order to maintain performance despite injury, players actively managed risks by using strategies like taping and taking painkillers (cf. 5). These tools were acknowledged as essential, particularly in later stages of their careers as a result of accumulated injuries (cf. 10). Hanna described her use of painkillers during tournaments as a legitimate risk management strategy: “Especially in a tournament like a World Championship, there's just such constant strain that pain is present. And to dampen it, especially for the important players or the game, I think it's actually okay.” Hanna's perspective illustrated the normalization of calculated risk-taking within elite handball (e.g., 5), where accepting and taking health risks was seen as an intrinsic part of success. However, the employed risk management strategies often revealed a paradox in that athletes managed immediate risks with tools like analgesics, which carry their own risks such as severe side effects (5). Nevertheless, the normalization of risk-taking, cultivated over years of competitive sport, shows how deeply ingrained the willingness to take physical risks was among athletes (e.g., 60, 61).

## General discussion

In this study, we adopted a constructivist lens to explore how elite handball players’ individually managed physical health risks and how this management evolved throughout their careers. This approach allowed us to interpret players’ experiences in the context of their career stages and personal development. Utilizing foundational theories of risk perception and risk management (29, 35), we investigated how athletes approached risk over time. Thereby, our study makes a novel contribution to the field by integrating frameworks on risk perception (28, 29) with the subsequent behavioral risk management strategies employed by elite athletes (22, 31, 60). While previous studies have often treated risk perception and evaluation separately from coping mechanisms or risky behaviors, the present study brings them together to provide a more comprehensive understanding of how athletes manage physical health risks over the course of their careers.

Furthermore, our focus on the athletes’ perspectives adds a critical layer to the understanding of risk management. By capturing the experiences of athletes, this study expands the knowledge on sociocultural mechanisms that were identified in previous research (e.g., 1, 7, 21). Our findings highlight that risk management is not merely a product of socialization or a response to external pressures but an active, self-regulated process in which athletes continuously reassess and refine their strategies over time. While building on sociological research on the culture of risk, which examines how institutional and social norms shape the normalization of risk-taking, this study extends the discussion by emphasizing the individual cognitive processes and strategic behavioral adaptations athletes develop throughout their careers. Thereby, the present study offers a nuanced view of how subjective risk perception shapes decision-making and health management in elite sports from the athlete's perspective.

Within our analysis, we constructed four patterns of physical health risk management among elite handball players: (1) *Externalizing risks and refraining from proactivity;* (2) *Relinquishing control under medical uncertainty;* (3) *Fluctuating prioritization of health or success;* (4) *Calculated health-risk taking to achieve success.* Overall, our four themes illustrate the fluid nature of risk management strategies throughout elite handball players’ careers. While these patterns were evident among athletes at specific points in their careers, not all athletes exhibited every pattern or went through them in the same order. However, employing a career-stage perspective enabled us to discern that certain risk management patterns were more prevalent at specific career stages.

Elite handball players’ strategies for managing physical health risks throughout their careers can be understood as existing on a spectrum, reflecting varying levels of awareness, control, and proactivity across the facets of risk management. On a passive dimension of the spectrum, athletes adopted a fatalistic approach, frequently *externalizing risks and refraining from proactivity*. Injuries were externally attributed to luck or fate. Consequently, particularly younger athletes did not perceive risks. Instead, they viewed injuries as arbitrary and unavoidable events that required no particular behavioral adjustments, reflecting cognitive elements that have been attributed to risk normalization and risk acceptance in previous studies (cf. 21, 22). A novel finding of this study is that, according to the Relational Theory of Risk (29), the causal relationship between risk objects (e.g., pain or physical strain) and objects at risk (e.g., injury) was not recognized by athletes. This lack of recognition further reinforced their passive approach to managing health risks.

In the second theme *complying with the norms by relinquishing control*, athletes were still passive in agency as they referred control over risk management to external authorities, such as coaches and medical professionals. The athletes demonstrated a limited awareness of the potential physical health risks, only acknowledging the existence of health threats when such concerns were validated by a formal diagnosis. This reliance on external judgement rather than self-monitoring extends findings in sports injury studies indicating that athletes frequently fail to perceive injury-related symptoms as serious unless validated by professionals (62).

At other times, athletes demonstrated a *fluctuating prioritization of health or success* based on situational pressures, such as the relevance of upcoming games or social and organizational factors (7, 9, 13, 34). These diverse influencing factors shaped which risks became most salient to the athlete at a given time and how athletes evaluated risks. When specific factors, such as the severity of injuries, heightened athletes’ awareness of the risk for potential sporting or medical consequences, athletes frequently re-evaluated their priorities and values. This ultimately shaped how athletes managed risks (31). This dynamic process aligns with the Relational Theory of Risk, showing that risk perception and evaluation evolve based on personal and contextual factors. This finding extends the culture of risk heuristics beyond a static, socialized response by highlighting the situational and temporal variability of athletes’ engagement with risk.

At the most active dimension of the spectrum, players perceived physical risks as an essential part of elite performance and exhibited *calculated health-risk taking to achieve success*. This strategy was characterized by a calculated risk-taking mindset; here, athletes acknowledged the potential for long-term consequences (21, 63) but willingly took risks by performing hurt or using analgesics to sustain peak performance. Particularly athletes at more advanced stages of their careers emphasized that their previous experiences with risk management allowed them to make informed choices about risk-taking, leading to increased risk-taking.

### Limitations and avenues for future research

Our career-perspective approach offered rich, longitudinal insights into individual risk management strategies. However, the study also has limitations in generalizing findings across sports or cultural contexts, as risk perceptions and management strategies are often shaped by sport-specific norms and changing organizational expectations (62). Future research could explore how these dynamics vary across different sports and cultural settings to identify broader patterns in risk perception and management. Comparative studies on sport-specific norms and organizational expectations may provide deeper insights into how athletes navigate physical health risks. Additionally, examining how evolving policies or medical advancements influence risk behaviors could further illuminate the adaptability of athletes’ strategies over time.

Furthermore, the utilized method of retrospective interviews could be supplemented with real-time data collection during or immediately following health events to capture more precise, contextually grounded insights. This approach could also reveal relevant discrepancies between immediate and long-term interpretations of physical health risks. Further research could also specifically integrate the perspectives of coaches and medical staff to facilitate a multi-dimensional view of the influence of team dynamics and medical protocols on athletes’ risk-related behavior.

### Practical implications

The results of this study indicate potential courses of action for organizations and stakeholders in elite sport, including coaches, sports psychologists and team doctors or external physicians. One key implication is the need for athlete-centered education programs that foster proactive physical health risk management. Programs should promote an internal locus of control, resilience, and awareness of long-term health risks (e.g., 64), empowering athletes to take an active role in injury prevention and recovery. Sport clubs and unions should facilitate access to independent health advisors, including general physicians and mental health professionals, fostering open dialogue about injury risks (20). Encouraging athletes to articulate their subjective perceptions to specialists outside of the sport network can enhance their awareness of the social and psychological factors shaping their risk evaluations and coping strategies. Integrating routine risk assessments and regular medical check-ins could further enhance athletes’ awareness of their physical limits. In this context, health monitoring software and periodic consultations could provide athletes with structured opportunities to assess concerns and refine health risk management strategies, which in turn could also encourage more proactive health management (cf. 26).

By showing that athletes’ risk perceptions often hinge on external validation, such as diagnoses, our study suggests that elite sports organizations must establish clear, transparent injury protocols that could give medical staff greater authority to make and communicate diagnoses that provide athletes with justification for rest periods when experiencing pain or illness (cf. 9, 57, 58). Regular health evaluations by independent medical professionals, alongside non-punitive injury reporting mechanisms, can help mitigate the fear of disclosing pain and injuries (34, 61). To prevent conflicts of interest for both athletes and medical professionals, organizations should implement policies ensuring medical staff's independence, such as mandating financial disclosure and removing performance-related incentives (9). These measures promote unbiased medical care, prioritizing athlete well-being over competitive demands (65).

## Data Availability

The datasets presented in this article are not readily available because restrictions apply to their availability, as they were used under ethical permission for the current study. Due to the sensitivity of health data, access is restricted to ensure compliance with data protection regulations and to safeguard participants’ privacy. Requests to access the datasets should be directed to Jlbursik@gmail.com.
